# Cadherin 6 Is a New RUNX2 Target in TGF-β Signalling Pathway

**DOI:** 10.1371/journal.pone.0075489

**Published:** 2013-09-12

**Authors:** Valentina Sancisi, Greta Gandolfi, Moira Ragazzi, Davide Nicoli, Ione Tamagnini, Simonetta Piana, Alessia Ciarrocchi

**Affiliations:** 1 Laboratory of Molecular Biology, Department of Oncology and Advanced Technologies, Azienda Ospedaliera Arcispedale S. Maria Nuova-IRCCS, Reggio Emilia, Italy; 2 Pathology Unit, Department of Oncology and Advanced Technologies, Azienda Ospedaliera Arcispedale S. Maria Nuova-IRCCS, Reggio Emilia, Italy; Sun Yat-sen University Medical School, China

## Abstract

Modifications in adhesion molecules profile may change the way tumor cells interact with the surrounding microenvironment. The Cadherin family is a large group of transmembrane proteins that dictate the specificity of the cellular interactions. The Cadherin switch that takes place during epithelial-mesenchymal transition (EMT) contributes to loosening the rigid organization of epithelial tissues and to enhancing motility and invasiveness of tumor cells. Recently, we found Cadherin-6 (CDH6, also known as K-CAD) highly expressed in thyroid tumor cells that display mesenchymal features and aggressive phenotype, following the overexpression of the transcriptional regulator Id1. In this work, we explored the possibility that CDH6 is part of the EMT program in thyroid tumors. We demonstrate that CDH6 is a new transforming growth factor-β (TGF-β) target and that its expression is modulated similarly to other EMT mesenchymal markers, both in vitro and in thyroid tumor patients. We show for the first time that CDH6 is expressed in human thyroid carcinomas and that its expression is enhanced at the invasive front of the tumor. Finally, we show that CDH6 is under the control of the transcription factor RUNX2, which we previously described as a crucial mediator of the Id1 pro-invasive function in thyroid tumor cells. Overall, these observations provide novel information on the mechanism of the EMT program in tumor progression and indicate CDH6 as a potential regulator of invasiveness in thyroid tumors.

## Introduction

The transdifferentiation of epithelial tumor cells towards a mesenchymal condition is a complex process that allows tumor cells to leave their original site and to invade adjacent tissues. During this transition (also known as epithelial-mesenchymal transition - EMT), the epithelial cells shed their differentiated characteristics, including cell-cell adhesion, polarity, and lack of motility, and acquire instead mesenchymal features, including motility, invasiveness, and resistance to apoptosis. The importance of the EMT program in mediating epithelial cancer progression is supported by a massive amount of evidence that has been published on this topic over the last 15 years [1-5]. While the relevance of this process to the biology of tumors is well established and fully accepted, the complexity of the molecular events and regulatory pathways at the basis of EMT is far from being fully understood. Furthermore, some of the molecular players involved in this process remain unknown. The first functional consequence of EMT program activation is the alteration of the epithelial tumor cell interactions with the surrounding microenvironment. The epithelial adhesion molecules, in particular the E-Cadherin, are displaced by the multiprotein complexes at the adherent junctions and are substituted by mesenchymal Cadherins (such as N-Cadherin). This alteration signals within the cells triggering the complicated cytoskeleton rearrangement that it is necessary to support cell motility [6]. The E-Cadherin and N-Cadherin are the prototypes and by far the most studied members of the large Cadherin family, which includes over 50 proteins in vertebrates and non-vertebrate organisms. Some evidence suggests that different Cadherins play non-redundant roles in cells and it is commonly believed that such large variability originates from the need of complex organisms to specifically differentiate intercellular interactions [7]. Despite this, the potential role of Cadherins other than the E- and N-Cadherin in cancer development and progression has being rarely investigated. Recently, we have shown that the aggressive phenotype induced by the transcription regulator Id1 in thyroid tumor cells is accompanied by acquisition of mesenchymal features and by deregulation of over 400 genes, most of which are known to be deregulated or partake in the EMT program. Among the Id1 target genes, the Cadherin-6 (CDH6, also known as K-Cadherin) was strongly induced by Id1 in thyroid tumor cells [8]. CDH6 is a class II Cadherin, which is expressed mainly in kidney and central nervous system [9-11]. CDH6 is highly expressed in renal tissue during embryogenesis in which it drives the mesenchymal-epithelial differentiation that is necessary for kidney morphogenesis [9,12,13]. In spite of its role in promoting the epithelial phenotype during embryogenesis, CDH6 has been described as strongly expressed in ovarian cancer and renal carcinoma [14,15]. In the latter, CDH6 expression has been shown to strongly correlate with aggressive tumor behavior and poorer patient outcome [16,17]. To date, nothing is known regarding the involvement of CDH6 in the EMT program during epithelial tumor development and progression. In this work, we investigated the ability of normal and tumor thyroid cells to activate the EMT program in response to transforming growth factor-β (TGF-β) and we explored the possibility that CDH6 is a TGF-β target during EMT in thyroid tumors. Intriguingly, we found that thyroid tumor cells constitutively display markers of an active EMT process as compared to normal thyrocytes, both in vitro and in human patients. We also showed that CDH6 is strongly induced by TGF-β treatment both in normal and tumor thyroid cells, and that its expression accompanies invasiveness in human thyroid tumor patients.

## Materials and Methods

### Cell cultures and treatments

B-CPAP, TPC1, and WRO human cell lines were obtained from Dr. Massimo Santoro, University of Naples (Naples, Italy) [18,19,20,21]. Nthy.ori 3.1 and FTC133 cell lines were purchased from Sigma-Aldrich (Milan, Italy). All cell lines were grown at 37°C/5% CO_2_. B-CPAP, TPC1, and WRO were grown in DMEM supplemented with 10% fetal bovine serum. Nthy.ori 3.1 were grown in RPMI supplemented with 10% fetal bovine serum. FTC133 were grown in DMEM:Ham’s F12 (1:1) supplemented with 10% fetal bovine serum. Id1- and RUNX2-overexpressing B-CPAP clones and control clones were grown in presence of 400 µg/ml geneticin (Life Technologies, Monza, Italy).

Cell lines were starved with culture medium containing 1% fetal bovine serum 16-18 hours before TGF-β treatment. TGF-β (Peprotech, Rocky Hill, NJ) was added in starvation medium at concentrations of 5 ng/ml and 100 ng/ml for the indicated periods of time.

For Actinomycin D (Sigma-Aldrich, Milan, Italy) and Cycloheximide (Sigma-Aldrich, Milan, Italy) treatments, cells were starved with 1% fetal bovine serum, were treated after 16-18 hours with Actinomycin D 5 mg/ml or cycloheximide 50 mg/ml for 4 hours, then TGF-β was added at 100 ng/ml for 24 hours.

For TGF-β inhibitor experiments, BCPAP and TPC1 cell lines were starved over night and treated with 10mM of SB-431542 (Abcam, Cambridge, UK) for 24h or 48h. For cells treated for 48h, fresh inhibitor was added after 24h by replacing the medium.

### RNA extraction from formalin-fixed paraffin-embedded tissues

Total RNA was collected from 5 slices, 5 µm thick, of formalin-fixed and paraffin-embedded PTCs using the High Pure RNA paraffin kit (Roche, Milan, Italy). All samples (normal, primary tumor, and metastasis) were manually dissected under microscopic guidance by two pathologists (MR and SP). In order to minimize the biases that a different tissue processing may introduce in RNA quality, we performed gene expression analysis comparing normal tissue, primary tumor, and metastasis from the same patient. Primary tumor and normal tissues were collected from the same slide. Quantity and purity of the total RNA were checked using the NanoDrop 2000 spectrophotometer (Thermo Scientific, Waltham, MA). Primers for qRT-PCR were designed in order to obtain amplicons less than 100 bp in length.

### RNA extraction and quantitative real time-PCR (qRT-PCR)

Total RNA purification from cells was performed with RNAeasy Mini kit (Qiagen, Milan, Italy). 500 ng of total RNA was retrotranscribed using the iScript cDNA kit (Biorad, Segrate, Italy). qRT-PCR was conducted using Sso Fast EvaGreen Super Mix (BioRad, Segrate, Italy) in the CFX96 Real Time PCR Detection System (BioRad, Segrate, Italy). Relative expression of target genes was calculated using the ΔΔCt method by normalizing to the geometric mean of three reference genes expression: Glyceraldehyde 3-phosphate dehydrogenase (GAPDH), Cyclophilin A (CYPA) and Beta-D-Glucuronidase (GUSB) unless otherwise specified. Sequences of primers are listed in Table S1.

### Western blot

For Western blot analysis, cells were lysed in RIPA buffer. Equal amounts of protein extracts were analyzed by SDS-PAGE using the Bio-Rad (Segrate, Italy) Mini-Protean apparatus. Staining was performed with the ECL Western blot detection reagent (GE Healthcare, Milan, Italy). Primary antibodies were mouse anti-E-CAD (BD Biosciences, Buccinasco, Italy), mouse anti-N-CAD (BD Biosciences, Buccinasco, Italy), rabbit anti-FN 1 (H-300, Santa Cruz Biotechnology, Santa Cruz, CA, USA), mouse anti-pERK (E-4, Santa Cruz Biotechnology, Santa Cruz, CA, USA), rabbit anti-pAKT (Santa Cruz Biotechnology, Santa Cruz, CA, USA), and mouse anti-Actin (AC-15, Sigma-Aldrich, Milan, Italy). For CDH6 we tried several antibodies from several dealers (Abcam ab64917 and ab71434, Santa Cruz sc-59974, Epitomics T0233). None of these antibodies gave reliable results in either western blot or immunohistochemistry. Secondary antibodies were HRP-conjugated anti-rabbit (GE Healthcare, Milan, Italy) and anti-mouse (GE Healthcare, Milan, Italy).

### Patient samples and immunohistochemistry

All PTC samples (N=15) and matched LNMs (N=7) were retrieved from the archive of the Pathology Unit of Arcispedale S. Maria Nuova. Description of the clinicopathological features of the analyzed PTC patients is provided in Table S2. Tissue specimens were fixed in 10% formalin, paraffin embedded, and cut in 4 µm thick sections. For the immunohistochemistry analysis, we used the mouse monoclonal anti-CDH6 antibody (HPA007047) from Sigma-Aldrich (Milan, Italy), which resulted in a strong membrane staining. As control of the specificity of the staining, we tested the antibody on renal carcinomas. No specific staining was observed in 3 mammary carcinoma samples. As indicated in the datasheet, this antibody does not work in western blot. Images were captured using a Nikon Eclipse E80 microscope (Nikon Instruments, Calenzano, Italy).

### Ethics statement

This project was approved by the “Comitato etico provinciale di Reggio Emilia” (local ethics committee of Reggio Emilia). Written informed consent was obtained by all the patients involved in this project. The local ethics committee approved the entire procedure of informed consent collection and patients data managing. This study has been conducted according to the Declaration of Helsinki.

### Immunofluorescence

Cells were grown in 4 well Lab-Tek Chamber slides (NunThermo Scientific, Waltham, MA, USA). After appropriate treatments, cells were fixed in 4% PFA in PBS 1X for 15 minutes at room temperature, permeabilized with 0.1% Triton in PBS 1X for 2 minutes, blocked with 20% FBS and 2% BSA in PBS 1X for 1 hour, then incubated with a mouse anti-Smad2/3 antibody (C-8, Santa Cruz Biotechnology, Santa Cruz, CA, USA) in a humidified chamber for 1 hour. Antibody binding was revealed with a secondary anti-mouse Alexa 488 conjugated antibody (Life Technologies, Monza, Italy). Cell nuclei were stained with DAPI (Life Technologies, Monza, Italy). Slides were mounted using the SlowFade mounting medium (Life Technologies, Monza, Italy) and observed using an Axiophot fluorescent microscope (Zeiss, Arese, Italy).

### Small interfering RNA (siRNA) transfection

Stealth RNA interference oligos against RUNX2 and control oligos were purchased from Life Technologies (Monza, Italy), and 30 nM of either anti-RUNX2 or control siRNA was transfected using the RNAiMax Lipofectamine reagent (Life Technologies, Monza, Italy) using the reverse transfection protocol.

### Statistical analysis

Statistical analysis was performed using GraphPad Prism Software (GraphPad, San Diego, California, USA). When statistical analyses were performed, the specific tests used are indicated in the figure legend.

Additional materials and methods are provided as supporting information.

## Results

### CDH6 is a new TGF-β target gene

**Figure 1:**
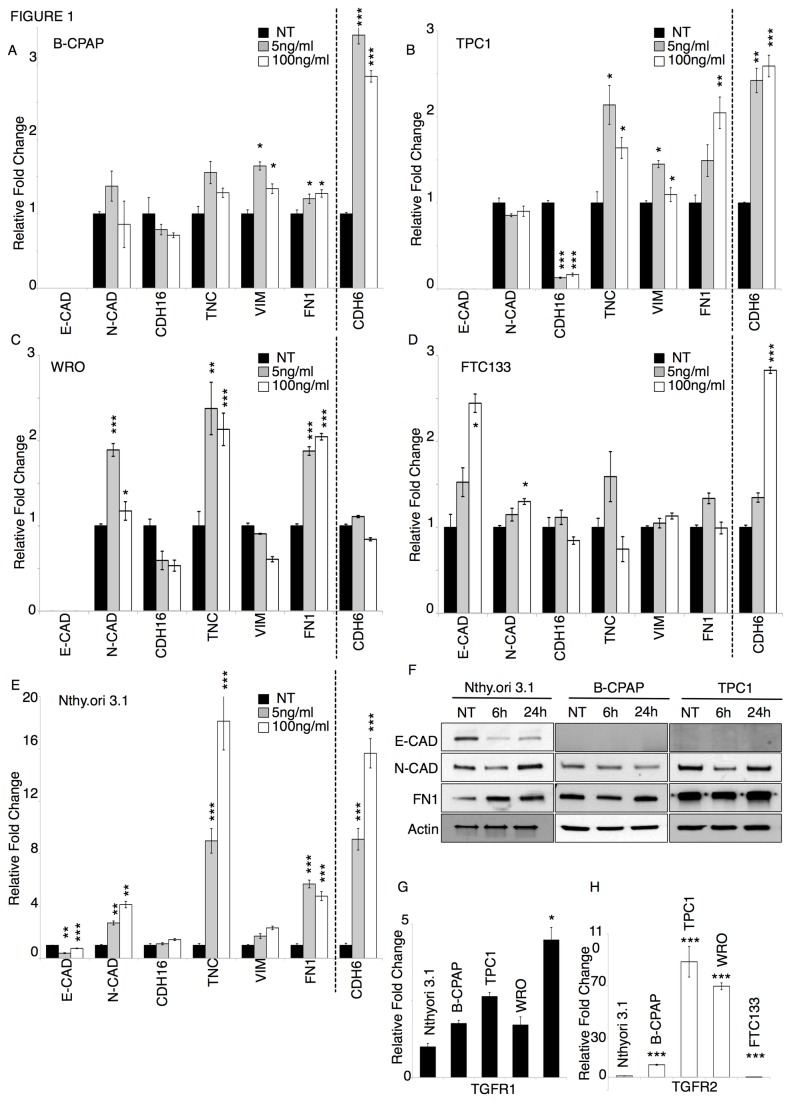
Nthy.ori 3.1 thyrocytes are more responsive to the TGF-β-mediated EMT program than tumor-derived cells. A-E) qRT-PCR analysis of EMT markers (E-CAD; N-CAD; CDH16; TNC; VIM; FN 1) and CDH6 in non-treated (NT; black bars) or TGF-β treated (5 ng/ml grey bars; 100 ng/ml white bars) thyroid-derived cell lines. The bars represent the average fold change of indicated genes in TGF-β treated cells as compared to non-treated cells, normalized to the geometric mean of levels of three reference genes: GAPDH, CYPA, GUSB. F) Western Blot analysis of E-CAD, N-CAD, FN 1, and Actin in Nthy.ori 3.1 cells, B-CPAP and TPC1 cells non-treated (NT) or treated with 100 ng/ml of TGF-β for 6h and 24h. G, H) qRT-PCR analysis of TGFR1 (G) and TGFR2 (H) in non-treated thyroid-derived cell lines. The bars represent the average fold change of TGFR1 and TGFR2 in tumor cells (B-CPAP, TPC1, WRO, and FTC-133) as compared to thyrocytes (Nthy.ori 3.1), normalized to the to the geometric mean of GAPDH, CYPA, GUSB levels. Error bars represent s.e.m. (n=3). p-value was calculated by two-tailed Student’s t-test. *** p≤ 0.001; ** p≤ 0.01; * p≤ 0.05. Each experiment has been replicated a minimum of two times with comparable results.

In order to investigate the effect of the TGF-β in thyroid tumor progression and the possibility that CDH6 is one of the TGF-β targets, five different thyroid-derived cell lines were selected and used as a model. The origin and specific features of the cell lines used are summarized in Table 1. Two different doses of TGF-β (5 ng/ml and 100 ng/ml) were given to cells for 24h, after which changes in the expression levels of established EMT markers and CDH6 were measured by means of qRT-PCR (Figure 1). Two epithelial markers - E-CAD and Cadherin-16 (CDH16) - and four mesenchymal markers - N-CAD, Tenascin C (TNC), Vimentin (VIM) and Fibronectin 1 (FN1) - were analyzed to monitor the activation of the EMT program [8,22-24]. All cell lines responded to TGF-β to some extent (Figure 1 A-E). However, the strength of the response was different between the Nthy.ori 3.1 thyrocytes and the tumor cell lines. In the Nthy. ori3.1 cells, E-CAD was significantly repressed, by the TGF-β treatment, while N-CAD, TNC, VIM, and FN 1 expression were considerably increased (Figure 1E). In contrast, TGF-β treatment induced only a modest effect on the four tumor-derived cell lines analyzed (Figure 1A-D). No E-CAD expression could be detected in any condition in B-CPAP, TPC1, and WRO cells, whereas CDH16 expression was detected in all cell lines and was significantly repressed only in TPC1 cells. Western Blot analysis of some of these markers in TGF-β treated Nthy.ori 3.1, B-CPAP and TPC1 cells confirmed the validity of the qRT-PCR results (Figure 1F). TGF-β treatment on the metastatic FTC133 cells resulted in increased E-CAD expression. This may be consistent with the need of metastatic cells to reverse the EMT phenotype necessary for metastatic site colonization [1]. Noticeably, CDH6 was strongly induced in all cell lines analyzed, with the exception of the WRO cells. While in tumor-derived cells CDH6 expression increased two-threefold after TGF-β treatment, in Nthy.ori 3.1 CDH6 induction was much more pronounced (eight-fifteen fold), similar to the one observed for TNC and superior to the induction observed for N-CAD and FN 1. These results demonstrate that CDH6 is a TGF-β target in thyroid cells and that its expression is modulated similarly to other mesenchymal markers. With the intent to understand whether this could be a thyroid specific mechanism, we have extended this analysis to other types of tumor. To this purpose, A549 lung cancer, A375 melanoma and MDA-MB-231 breast cancer cell lines were treated with TGF-β and expression of CDH6 was monitored by qRT-PCR. We were not able to detect CDH6 expression in either A375 or MDA-MB-231 cell lines (data not shown). In A549 lung cancer cell line CDH6 was expressed and induced upon TGF-β treatment at levels comparable to the thyroid tumor cell lines (Figure S1). These results suggest that CDH6 expression is tissue specific and the ability of TGF-β to target this gene is common to other types of tumor.

**Table 1:** Origin and mutational status of thyroid-derived cell lines.

**Cell line**	**Nthy.ori 3.1**	**B-CPAP**	**TPC1**	**WRO**	**FTC133**
Origin	Normal thyroid	PTC	PTC	FTC	FTC-LNM
BRAF	nd	V600E	wt	wt/V600E*	wt
NRAS	nd	wt	wt	wt	wt
HRAS	nd	wt	wt	wt	wt
KRAS	nd	wt	wt	wt	wt
PI3KCA	nd	wt	wt	wt	wt
PTEN	nd	wt	wt	wt	R130stop
TP53	nd	D259Y	wt	wt	R273H
RET/PTC1	nd	-	+	-	-
RET/PTC3	nd	-	-	-	-
PAX8/PPARg	nd	-	-	-	-

PTC, Papillary Thyroid Carcinoma; FTC, Follicular Thyroid Carcinoma; FTC-LNM, Lymph Node Metastasis from Follicular Thyroid Carcinoma; nd, not determined; wt, wild-type; * BRAF V600E mutation in WRO cell line is reported only by some authors; + presence of rearrangement; - absence of rearrangement (modified from Saiselet et al. 2012) [21].

All together, our observations suggested that tumor-derived cells are less responsive to the TGF-β than normal thyrocytes. A lower expression of the TGF-β receptors in thyroid tumor cells could result in a minor capacity to transduce the TGF-β signal. To test this hypothesis, we compared the expression levels of the TGF-β receptors 1 and 2 in the five cell lines by qRT-PCR analysis. As shown in Figure 1G and H, all cell lines displayed comparable levels of the TGFR1, while a more heterogeneous expression of the TGFR2 was observed. However, in contrast with the extent of the TGF-β response, all tumor-derived cells, with the exception of the FTC133 cells, had higher levels of the TGFR2 than the Nthy.ori 3.1 cells.

### The EMT program is constitutively active in tumor-derived cells.

**Figure 2:**
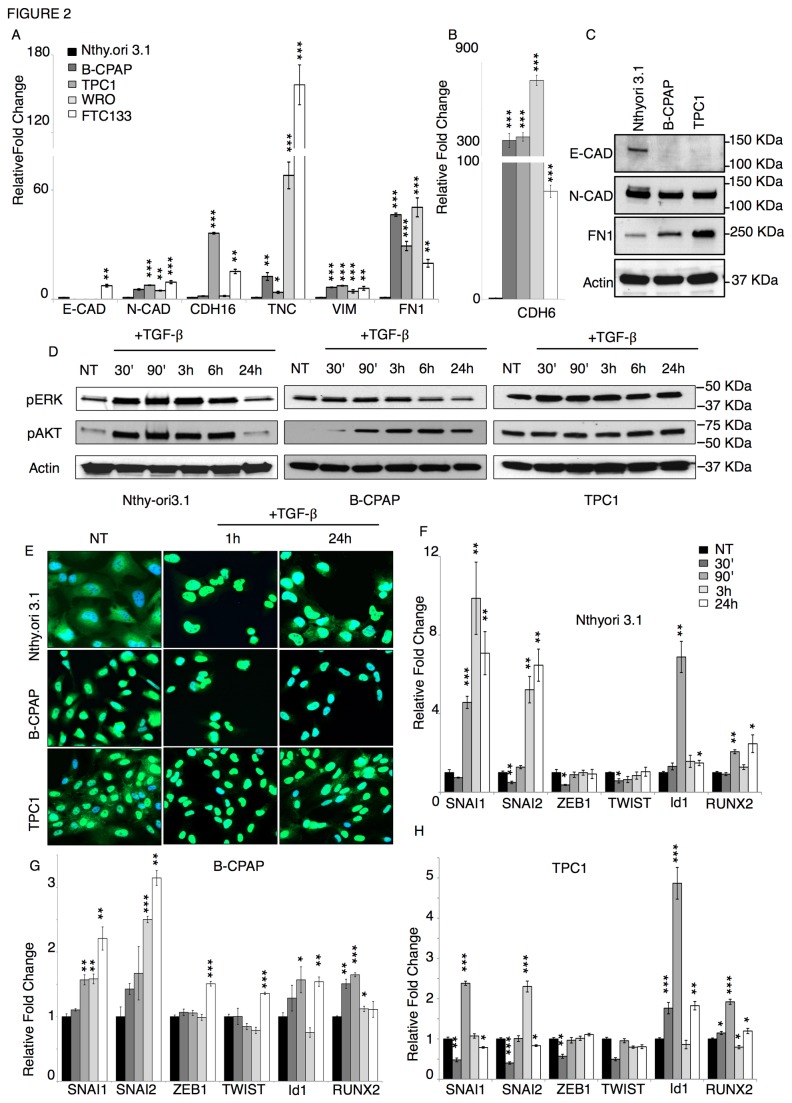
Tumor-derived cell lines display a constitutive EMT-like phenotype. qRT-PCR analysis of EMT markers (A) (E-CAD; N-CAD; CDH16; TNC; VIM; FN 1) and CDH6 (B) in non-treated thyroid-derived cell lines. The bars represent the average fold change of the indicated genes in tumor cells (B-CPAP, TPC1, WRO, and FTC-133) as compared to thyrocytes (Nthy.ori 3.1), normalized to the geometric mean of levels of three reference genes: GAPDH, CYPA, GUSB. C) Western Blot analysis of E-CAD, N-CAD, FN1 and Actin in non-treated Nthy.ori 3.1; B-CPAP and TPC1 cells. D) Western Blot analysis of phosphorylated ERK, phosphorylated AKT, and Actin in Nthy.ori 3.1 (Left panels); B-CPAP (middle panels) and TPC1 (right panels) cells untreated (NT) or after TGF-β exposure for the indicated times. E) Immunofluorescence staining of SMAD2/3 proteins (green) in Nthy.ori 3.1 (upper panels); B-CPAP (middle panels) and TPC1 (lower panels), non-treated (NT) or after TGF-β exposure for the indicated times. DAPI (Blue) stains the nuclei. Magnification 200X. F-H) qRT-PCR analysis of transcription factors known to partake in the EMT program (SNAI1, SNAI2, ZEB1, TWIST, Id1, and RUNX2) in Nthy.ori 3.1 (E), B-CPAP (F) and TPC1 (G) cells, non-treated (NT) or treated with TGF-β for the indicated times. The bars represent the fold change of the indicated genes in TGF-β treated cells as compared to the non-treated cell levels, normalized to the geometric mean of GAPDH, CYPA, GUSB levels. p-value was calculated by two-tailed Student’s t-test. *** p≤ 0.001; ** p≤ 0.01; * p≤ 0.05. Error bars represent s.e.m. (n=3).

We investigated the possibility that tumor-derived cells have already undergone an EMT transformation by comparing the expression levels of the above-mentioned EMT markers between normal Nthy.ori 3.1 thyrocytes and tumor-derived cells. Noticeably, the expression of all mesenchymal markers analyzed (N-CAD, TNC, VIM and FN 1) was significantly higher in the tumor cells than in the Nthy.ori 3.1 thyrocytes. By contrast, E-CAD expression could be detected only in the Nthy.ori 3.1 and FTC133 cells, while no expression of this epithelial marker was detected in the other tumor-derived cells (Figure 2A). CDH16 expression was higher in TPC1 and FTC133 than in the rest of the cell lines. Noticeably, CDH6 expression was greatly enhanced in all thyroid tumor-derived cells as compared to the normal thyrocytes (Figure 2B). Western blot analysis was in accordance with the gene expression data (Figure 2C). Then we tested whether the EMT-like phenotype displayed by thyroid tumor cells was accompanied by a constitutive activation of the TGF-β downstream pathways. TGF-β signals within the cells through three major pathways: nuclear translocation of the SMAD2/3 proteins, the activation of the MAPK, and of PI3K cascade [25]. Since our interest was mainly on the mechanism controlling the biology of papillary thyroid tumors, we limited the analysis to papillary-derived tumor cells. Nthy.ori 3.1, B-CPAP, and TPC1 cells were treated with TGF-β and changes in the activation of the three signaling pathways were monitored. In the Nthy.ori 3.1 thyrocytes, the levels of both pERK and pAKT rose early after the addition of TGF-β. In B-CPAP cells, pERK levels did not change in response to TGF-β, while AKT phosphorylation was significantly induced. In TPC1 cells, phosphorylation levels of both ERK and AKT did not change in response to TGF-β (Figure 2D). Furthermore, in untreated Nthy. ori.3.1 cells SMAD2/3 were localized in the cytoplasm, to be strongly translocated to nuclei early after TGF-β exposure (Figure 2E upper panels). In untreated B-CPAP cells SMAD2/3 were localized mainly in the nuclei and their localization did not change significantly in the presence of TGF-β (Figure 2E middle panels). In untreated TPC1 cells, the SMAD2/3 staining was visible in both nucleus and cytoplasm. However, after TGF-β treatment, the cytoplasmic staining was no longer detectable, indicating that all the protein was translocated to the nuclei (Figure 2E lower panels). Overall, these data suggest that TGF-β-dependent signaling cascades are constitutively active in thyroid tumor cells. These results may be partially explained by the fact that tumor cells, including the B-CPAP and TPC1, often harbor somatic mutations in components of these signaling pathways (see Table 1). However, tumor cells retain a partial ability to respond to the TGF-β signal by further activating some of these pathways, like AKT in the B-CPAP and SMAD2/3 in the TPC1. The EMT program is controlled by a number of transcription factors, which are known targets of TGF-β [5,26]. Both Id1 and RUNX2 have been shown to be TGF-β targets and have been proposed as crucial regulators of the EMT program [8,27-29]. We investigated how the expression of EMT-related transcription factors changed in response to TGF-β in thyroid cell lines. SNAI1, SNAI2, Id1, and RUNX2 were induced by TGF-β in both Nthy.ori 3.1 and TPC1 cells. In accordance with what was observed for the EMT markers (Figure 1), the activation of SNAI1, SNAI2, and Id1 was lower in TPC1 than in Nthy.ori 3.1 (Figure 2F-H). In B-CPAP cells slight change in SNAI1, SNAI2, ZEB1 and TWIST expression was observed after 24h (Figure 2G). ZEB1 and TWIST levels were not affected in Nthy.ori 3.1 and TPC1 cells. It is worth pointing out that the induction of Id1 and RUNX2 preceded the activation of the other factors, suggesting that these factors are early targets of the TGF-β-dependent EMT program.

### CDH6 is overexpressed in human PTCs and mainly localized at the invasive front of the tumors.

**Figure 3:**
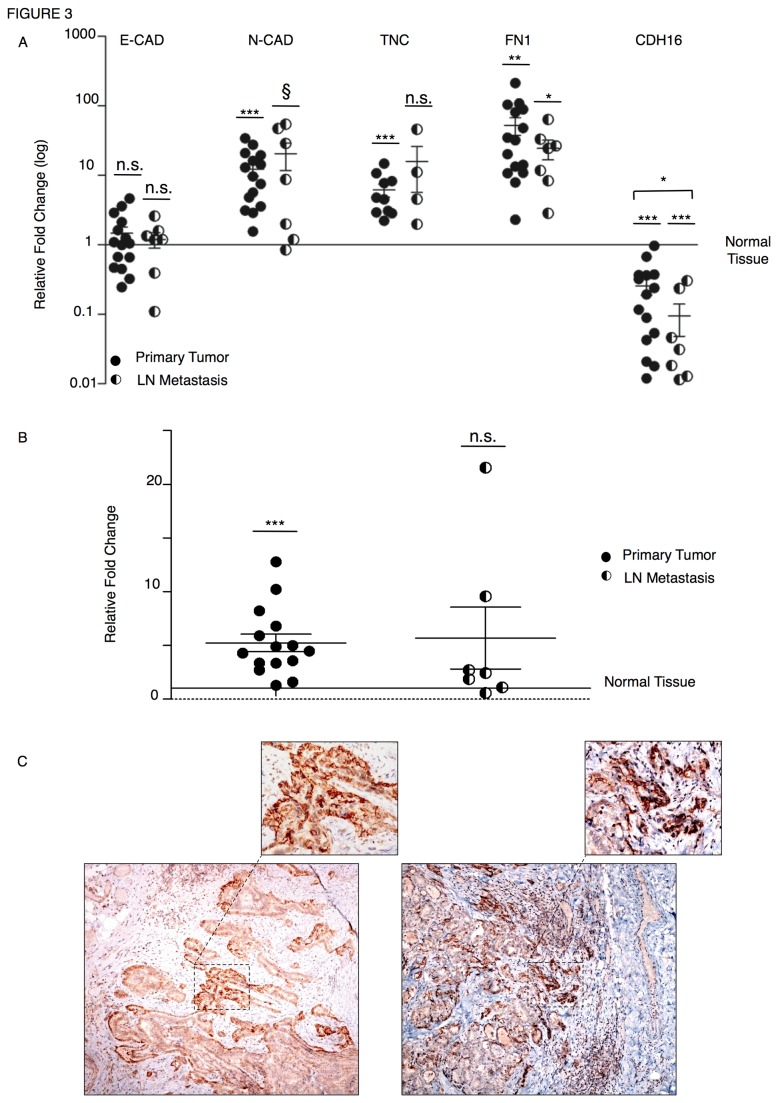
Primary and metastatic thyroid tumor cells display a higher expression of EMT-markers than normal thyroid tissue. A) Scatter plot representation of the relative fold expression of EMT markers (E-CAD, N-CAD, TNC, FN 1, and CDH16) obtained by qRT-PCR in primary tumors (n=15; black circles) and LNMs (n=7; black and white circles) as compared to the respective normal tissue (baseline) from human PTC patients. We were not able to obtain detectable levels of TNC for 5 PTC samples and 2 LNMs. B) Scatter plot representation of the relative fold expression of CDH6 obtained by qRT-PCR in primary tumor (n=15; black circles) and LNMs (n=7; black and white circles) as compared to the respective normal tissue (baseline) from human PTC patients. Target gene expression (A and B) was normalized to the geometric mean of GAPDH, CYPA, GUSB levels. p-value was calculated by two-tailed Student’s t-test. *** p≤ 0.001; ** p≤ 0.01; * p≤ 0.05. C) Representative immunohistochemistry analysis of CDH6 expression (brown) in two PTC samples. A total of 15 PTC samples were analyzed. Invasive front is defined as the boundary between tumor lesion and non-neoplastic thyroid tissue. Hematoxylin (blue). Magnification 100X. The insets show higher magnification of the same field and represent nest of cells infiltrating the tumor capsule. IHC staining was analyzed by light microscopy by two authors (AC, SP). Magnification 200X.

To confirm in vivo that thyroid tumor cells display features of a constitutively active EMT-program, we analyzed the expression of EMT markers in human papillary thyroid carcinoma (PTC) samples and matched lymph node metastases (LNMs). We collected total RNA from tumor and surrounding normal tissue for 15 PTC patients and we analyzed EMT markers by means of qRT-PCR (Figure 3A). Noticeably, the expression of mesenchymal markers (N-CAD, TNC, and FN 1) was significantly higher in the vast majority of tumors (black circles) than in normal tissues (baseline). By contrast, CDH16, marker of the epithelial phenotype, was significantly less expressed in tumor cells than in normal thyrocytes. E-CAD expression was not significantly different in tumor sample as compared with the normal tissue. LNMs were available for 7 of the analyzed PTC samples. The expression pattern of EMT markers in LNM (black and white circles) reflected the trend observed in the primary lesions, confirming that active EMT features are a constitutive hallmark of tumor transformation in thyroid tumors. To confirm that CDH6 is a marker of the mesenchymal phenotype in thyroid tumors, we investigated the expression of CDH6 in the aforementioned PTC samples by qRT-PCR (Figure 3B). As for the other mesenchymal markers, CDH6 expression was higher in tumor tissue (black circles) and LNMs than in normal tissue (baseline). The CDH6 expression levels seemed to decrease in the transition from primary to metastatic site, suggesting that this factor may be required particularly in the early phases of the metastasization process. Next, we analyzed the CDH6 protein expression by immunohistochemistry in the same PTC samples previously analyzed by qRT-PCR. As expected, CDH6 expression was clearly higher in tumor cells than in normal tissue (Figure 3C and Figure S2A-F). However, the expression of CDH6 was not uniform within the tumor but was restricted to groups of cells. Intriguingly, CDH6-positive cells were localized preferentially at the invasion front of the tumor, strongly supporting the hypothesis that CDH6 is important in controlling cell motility and invasiveness in PTCs (Figure 3C and Figure S2A and B).

### CDH6 splicing variants display a different profile in thyroid tumor-derived cells

**Figure 4:**
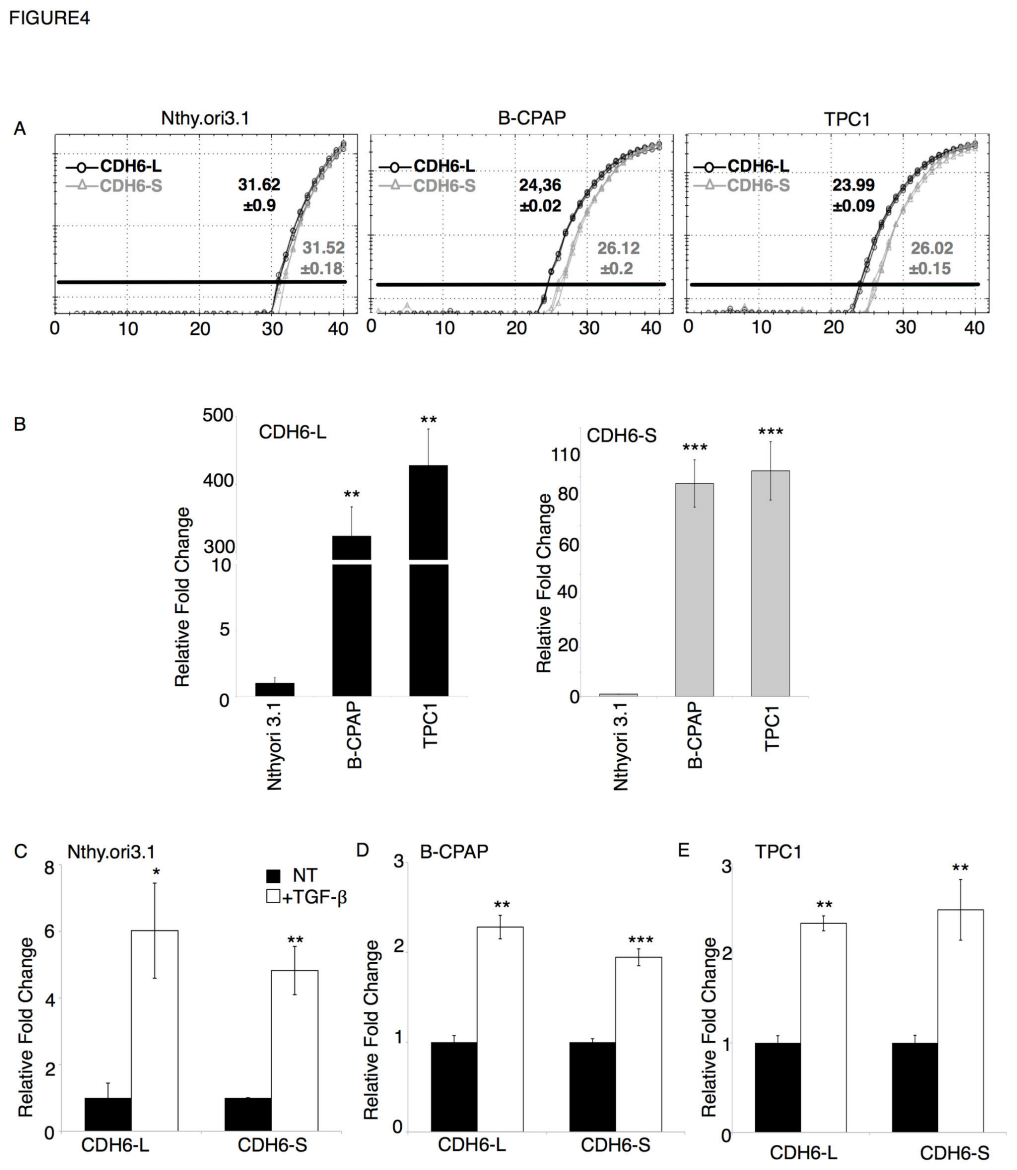
Analysis of the expression levels of CDH6 splicing variants in thyroid-derived cells. A) Representative amplification curves of CDH6-L (long isoform; black line) and CDH6-S (short isoform, gray line) in non-treated Nthy.ori 3.1 (left panel), B-CPAP (middle panel) and TPC1 (right panel) obtained by qRT-PCR amplification. Numbers represent the average Ct value +/- s.e.m. of a triplicate amplification. B) qRT-PCR analysis of CDH6-L (left) and CDH6-S (right) in non-treated thyroid-derived cell lines. The bars represent the average fold change of the indicated genes in B-CPAP and TPC1 as compared to Nthy.ori 3.1. Target genes were normalized to the geometric mean of levels of three reference genes: GAPDH, CYPA, GUSB. C-E) qRT-PCR analysis of CDH6-L and CDH6-S in non-treated (NT; black bars) or TGF-β treated (white bars) Nthy.ori 3.1 (C), B-CPAP (D), and TPC1 cells (E). The bars represent the average fold change of the CDH6 variants in TGF-β treated cells as compared to non-treated cells, normalized to the geometric mean of GAPDH, CYPA, GUSB. p-value was calculated by two-tailed Student’s t-test. *** p≤ 0.001; ** p≤ 0.01; * p≤ 0.05.

**Figure 5:**
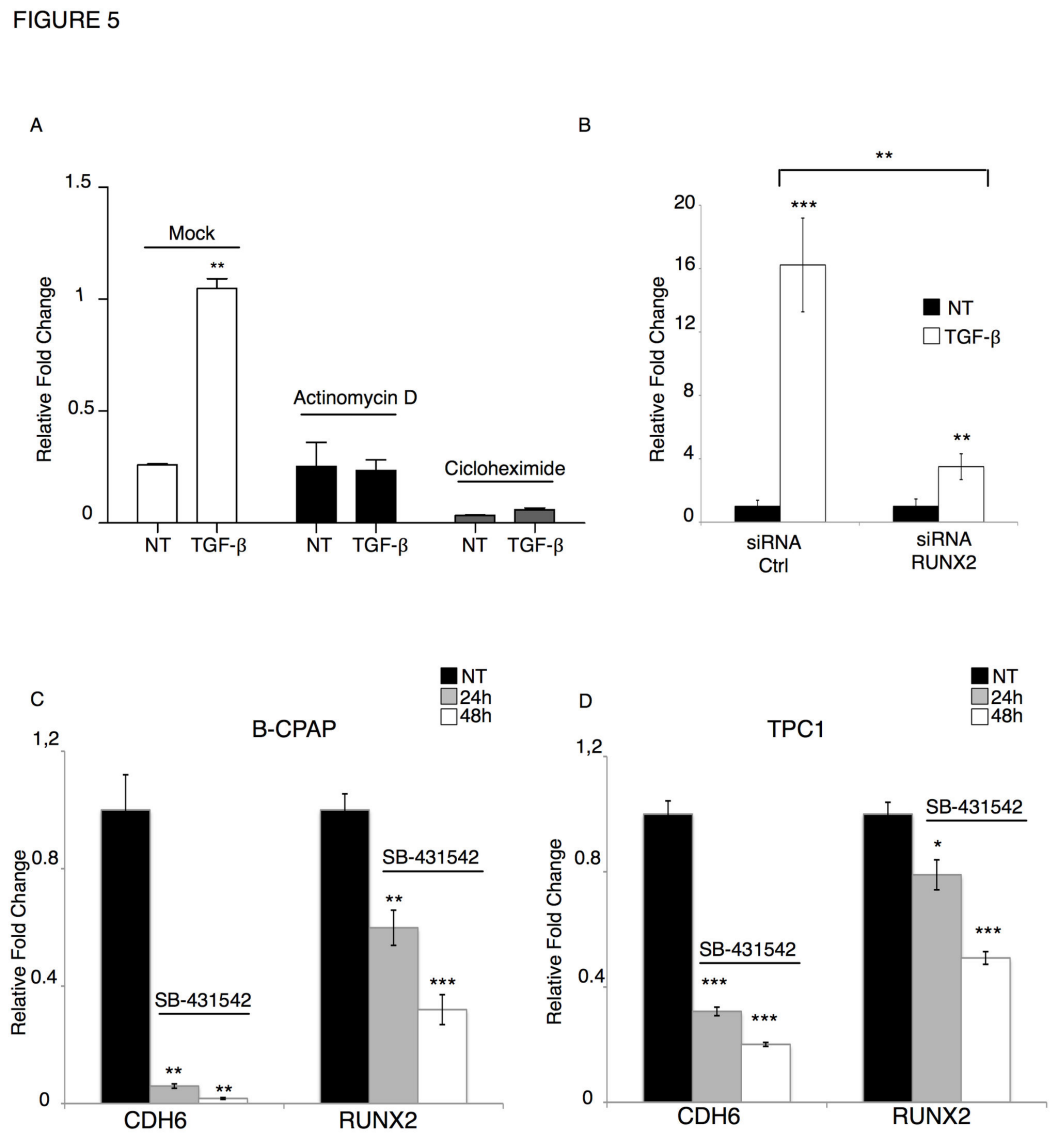
TGF-β dependent CDH6 induction is mediated by RUNX2. A) qRT-PCR analysis of CDH6 levels in non-treated (NT) and TGF-β treated (TGF) Nthy.ori cells, in presence of DMSO (mock, white bars), Actinomycin D (grey bars), cycloheximide (black bars). The bars represent the fold expression of the CDH6 mRNA in the indicated samples normalized to the GAPDH levels. The results are the average of two different replicates. B) qRT-PCR analysis of CDH6 levels in Nthy.ori 3.1 cells non-treated (black bars) or treated with TGF-β (white bars) after transfection with RUNX2 siRNA (right) or control siRNA (left). For both control siRNA and RUNX2 siRNA-treated samples, the bars represent the relative fold change of CDH6 upon TGF-β treatment as compared to non-treated cells. The graphs show one representative experiment. The experiment was replicated three times, obtaining comparable results. C and D) qRT-PCR analysis of RUNX2 and CDH6 mRNA levels in B-CPAP (C) and TPC1 (D) cells non-treated (NT, black bars) or treated with 10mM SB-431542 for 24h (grey bars) or 48h (white bars). Expression levels of CDH6 and RUNX2 were normalized to the geometric mean of GAPDH and CYPA. p-value was calculated by two-tailed Student’s t-test. *** p≤ 0.001; ** p≤ 0.01; * p≤ 0.05.

The *CDH6* gene codes for two different splicing isoforms, starting from a common promoter. The long isoform comprises both an extracellular domain (containing 4 ectodomain modules –EC– and a membrane proximal extracellular domain–MPE) and a cytoplasmic domain (containing the membrane proximal conserved domain–MPC- and the conserved catenin binding site–CBS) [7] (Figure S2G). Instead, the short isoform displays only the extracellular domain but lacks the cytoplasmic domain. Due to the ability of transmembrane proteins to transduce the signals within the cell through their cytoplasmic domains, it is reasonable to suppose that the two variants may play different roles. To address this issue, we analyzed the expression of the two CDH6 isoforms by qRT-PCR using specific primers (Figure S2G). Both isoforms were expressed in all cell lines (Figure 4A) and more strongly expressed in tumor-derived cells than in Nthy.ori 3.1 (Figure 4B), as observed for the total CDH6 (Figure 2B). Intriguingly, the expression change in tumor-derived cells (B-CPAP and TPC1) as compared to the Nthy.ori 3.1 was more pronounced for the CDH6-long isoform (CDH6-L) than for the short isoform (CDH6-S). Further, we analyzed changes in the expression levels of CDH6-L and CDH6-S isoforms by TGF-β treatment. Both isoforms were similarly induced by TGF-β in all cell lines (Figure 4C, D, E). This suggests that TGF-β controls CDH6 expression at the transcriptional level. To confirm this hypothesis we investigated the effect of TGF-β treatment on CDH6 expression in the absence of active transcription (with Actinomycin D) or in the absence of active protein synthesis (with Cycloheximide). As shown in Figure 5A, the addition of Actinomycin D completely abolished the TGF-β-mediated CDH6 induction, confirming that the regulatory effect of TGF-β requires an active transcription. Furthermore, the addition of Cycloheximide also resulted in a complete suppression of TGF-β induction, demonstrating that the effect on the CDH6 expression is indirect and requires the previous synthesis of an activator factor.

### RUNX2 mediates the TGF-β effect in thyroid cells

In a recent microarray analysis, we found CDH6 as a target of Id1 in aggressive thyroid tumor cells [8]. Further, we showed that the transcription factor RUNX2, also a target of Id1, is a crucial mediator of the Id1 pro-invasive function in thyroid tumor cells [30]. Both RUNX2 and Id1 have been proposed to control EMT in epithelial tumors. We reasoned that RUNX2 could be responsible for the TGF-β-dependent CDH6 induction. To test this hypothesis, Nthy.ori 3.1 cells were transfected with siRNA against RUNX2 or control siRNA and treated with TGF-β for 24h. As shown in Figure 5B, the siRNA-mediated RUNX2 depletion profoundly impaired the TGF-β effect on CDH6 expression. These experiments confirm that CDH6 is a target of RUNX2 in the TGF-β pathway. However, siRNA mediated RUNX2 ablation does not completely abolish the TGF-β effect on CDH6 expression suggesting that other TGF-β–dependent factors beside RUNX2 may be involved in controlling this gene in thyroid tumor cells.

RUNX2 has been shown to control the expression of SNAI factors [29]. After TGF-β treatment, RUNX2 induction occurs earlier that SNAI1 and SNAI2 activation. Thus, it is possible that the effect of RUNX2 on CDH6 expression could be indirect and mediated by SNAI factors. However, RUNX2 silencing does not affect TGF-β- dependent SNAI1 or SNAI2 induction suggesting that these transcription factors are not involved in this regulation (Figure S2H-I).

We showed that TGF-β signaling pathway is constitutively activated in thyroid tumor cells and suggested that RUNX2 and CDH6 is part of this pathway. If this hypothesis is correct blocking the TGF-β signaling in thyroid cancer cells should affect RUNX2 and CDH6 expression levels. To address this issue we treated B-CPAP and TPC1 cells with SB-431542, a small molecule known to block kinase activity of the TGF-β receptors family [30]. Noticeably, exposure to SB-431542 results in a strong repression of CDH6 and RUNX2 in both B-CPAP and TPC1 cells, which was clearly visible after 24h and increased after 48h from the beginning of the treatment (Figure 5C,D). The inhibitory effect of the compound was stronger on CDH6 expression than on RUNX2 expression. This suggests that other pathways cooperate with the TGF-β signals in controlling RUNX2 expression in thyroid tumor cells.

**Figure 6:**
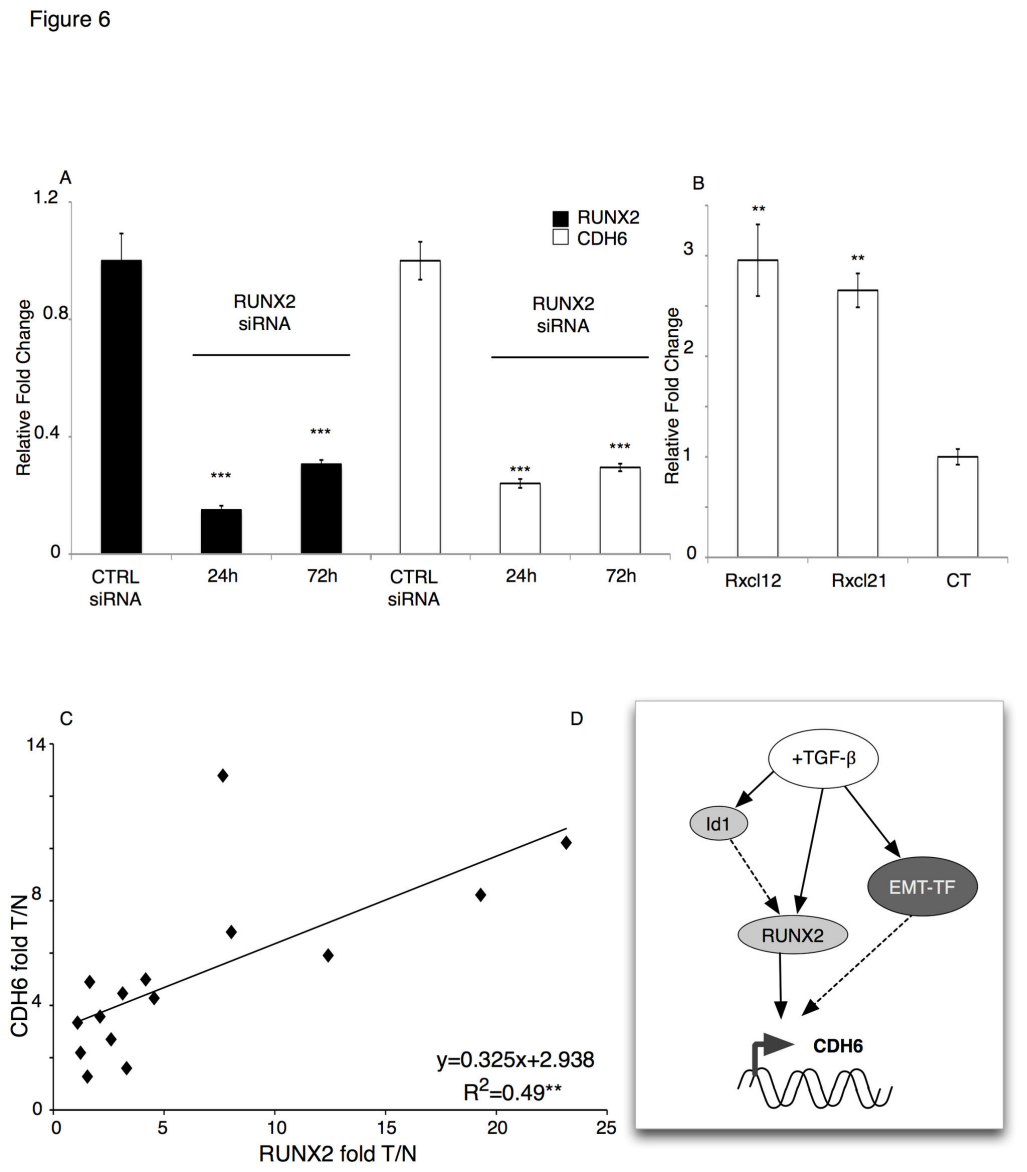
CDH6 is a new RUNX2 target in thyroid tumor cells. A) qRT-PCR analysis of RUNX2 (black bars) and CDH6 (white bars) mRNA levels in Id1-overexpressing cells (Id1A) upon RUNX2 siRNA transfection (24 hours and 72 hours after siRNA transfection). The bars represent the average fold change of indicated genes in cells transfected with RUNX2 siRNA as compared to cells transfected with control siRNA, normalized to the GAPDH levels. B) qRT-PCR analysis of CDH6 levels in cells stably overexpressing RUNX2 (Rx12 and Rx21) and control cells (CT). The bars represent the average fold change of CDH6 in RUNX2 overexpressing cells as compared to control cells, normalized to the GAPDH levels. Control represents the average CDH6 expression in two different stable clones transfected with an empty vector (Ctrl 4N and Ctrl 5N). Id1A, Rx12, Rx21, Ctrl4N, and Ctrl5N were previously described (26). p-value was calculated by two-tailed Student’s t-test. *** p≤ 0.001; ** p≤ 0.01; * p≤ 0.05.C) Linear regression analysis of the relative fold expression of CDH6 (Y-axis) and RUNX2 (X-axis) in primary tumors (n=11) as compared to the respective normal tissue from human PTC patients. CDH6 and RUNX2 levels were normalized to the geometric mean of GAPDH, CYPA and GUSB. Correlation was statistically significant (R^2^=0.4985; p-value= 0.0033). T/N= fold change Tumor vs Normal. D) Schematic representation of the TGF-β pathway in controlling CDH6 expression. TGF-β signaling induces activation of RUNX2 that in turn control CDH6 expression. Our data seem to suggest that other TGF-β dependent mechanisms, involving other transcription factors (EMT-TF) cooperate with RUNX2 in controlling CDH6 expression. We showed that CDH6 is a target of Id1 in thyroid tumor cells with aggressive phenotype. In this work we showed that the Id1 dependent CDH6 induction is mediated by RUNX2. However, since both RUNX2 and Id1 are early target genes of the TGF-β signaling we hypothesize that TGF-β controls RUNX2 expression independently by Id1.

We recently showed that RUNX2 is a crucial mediator of the Id1 aggressive phenotype in thyroid tumor cells [31]. Thus, to further confirm that RUNX2 controls the expression of CDH6 we used a B-CPAP-derived cell line stably expressing Id1 [8,31], in which we had previously reported that CDH6 level was significantly induced. RUNX2 siRNA transfection in Id1 overexpressing cells resulted in a profound reduction of the CDH6 expression (Figure 6A). Then we investigated the expression of CDH6 in two B-CPAP-derived cell lines stably expressing RUNX2 (Rx12 and Rx21) [31]. As shown in Figure 6B, CDH6 level was significantly higher in RUNX2 overexpressing cells than in control cells. Overall, these data demonstrate that CDH6 expression is controlled by RUNX2 in thyroid tumor cells. Intriguingly, CDH6 expression is not affected by transient overexpression of RUNX2 in Nthy.ori 3.1 cells (Figure S3B, C). This could imply that a stable expression of RUNX2 is necessary to lead to a constitutive activation of CDH6. In alternative, it is possible that the ability of RUNX2 of controlling this gene is context dependent and linked to other signals, as already reported for other RUNX2 target genes [31].

We showed that CDH6 (Figure 3B-C) is overexpressed in human PTCs, as we previously reported for RUNX2 [31]. Thus we investigated the existence of a correlation between RUNX2 and CDH6 expression in PTC samples. Strikingly, regression analysis showed a strong correlation in the expression patterns of the two genes in the PTC samples analyzed (n=15) (Figure 6C). Overall these data demonstrate that CDH6 is a novel RUNX2 target gene. Based on these observations we can hypothesize a model in which RUNX2 is the major mediator of TGF-β signaling in controlling the expression of CDH6 in thyroid tumor cells (Figure 6D).

## Discussion

**Figure 7:**
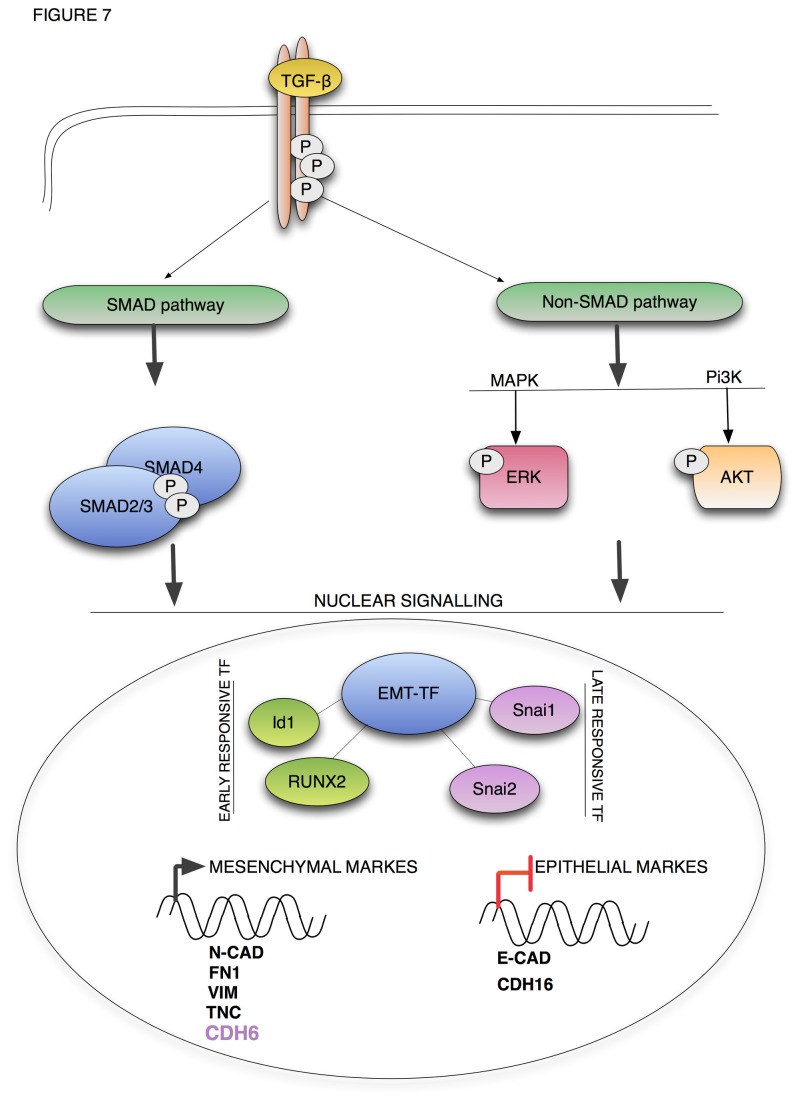
TGF-β signaling in EMT program in thyroid cells. Schematic representation of the TGF-β dependent EMT program in thyroid cells. Malignant transformation of thyroid cells is accompanied by a constitutive activation of the TGF-β signaling pathway. TGF-β cascade controls the expression of a number of transcription factors (EMT-TF) including RUNX2 that alter the gene expression profile of the cells to activate the EMT program.

The EMT program is controlled by a number of extracellular signals and is supported by the activation of a precise pattern of transcription factors that induce a complete re-programming of the cellular phenotype by changing the transcriptional profile of the cell. In spite of the extensive knowledge on this process that has been gained over the years, the molecular complexity of EMT still seems far from being fully unraveled. In this work, we show for the first time that CDH6 is a novel TGF-β target gene in thyroid cells, and we demonstrate that its expression is controlled by RUNX2, which we have recently shown to be an important mediator of aggressive phenotype in thyroid tumor cells (Figure 7) [31]. RUNX2 overexpression has been described in thyroid carcinomas [32] and has been linked to the EMT process in different types of tumors [27,29,33]. With this work we provide an additional piece of information concerning its function in this process. CDH6 is a type II Cadherin, which has been implicated during embryogenesis in the morphogenesis of the central nervous system and of the kidney [9-11]. In renal development, the CDH6 controls the phenotypical conversion of mesenchymal precursors into the epithelium, which will constitute the developing nephrons [9,12,13]. This is intriguing since our data would suggest that in a pathological setting such as tumor development, the CDH6 plays an opposite role. We have previously shown that CDH6 is strongly induced in thyroid tumor cells with an aggressive phenotype and presents mesenchymal features [8]. Here we show that by TGF-β treatment CDH6 is induced, like other well-established mesenchymal markers, including N-CAD and FN 1. Further analyses are required in order to fully understand the functional relevance of CDH6 in the EMT program during tumor progression. However, the fact that CDH6 levels increase in response to TGF-β as the N-CAD and in contrast with the downregulation of E-CAD suggests that this protein is involved in the Cadherins switch, favoring the rupture of the tight cell-cell junctions and increasing the motility of tumor cells. This hypothesis is strongly supported by our observation that in human PTC samples, CDH6 expression is detectable mainly at the invasive front of the tumor, where tumor cells need to loosen their intracellular interaction to gain motility and invade the adjacent tissues.

To our knowledge, this is the first report showing that CDH6 is expressed in thyroid carcinomas. In line with these observations, Puxeddu et al have reported that the imposed overexpression of the RET/PTC3 oncogene in normal thyrocytes results in a significant up-regulation of CDH6 and FN 1 [34], supporting the idea that CDH6 is turned on during thyrocyte transformation and that its expression follows the trend of other mesenchymal markers. Our data also show that CDH6 expression is constitutively higher in tumor cells than in normal thyrocytes, both in vitro and in vivo. This is in line with what has been reported in ovarian cancer and renal carcinomas, where an overexpression of CDH6 is observed in tumor cells as compared with the normal counterpart [14,15]. In renal carcinomas, where CDH6 expression is considered an established tumor biomarker, higher levels of CDH6 are prognostic of tumor aggressiveness and poor outcome [16,17]. This is in accordance with our observation that CDH6 is expressed mainly in thyroid tumor cells with an aggressive phenotype and localized at the invasive front of PTCs, and supports the idea that CDH6 may favor the spreading of tumors by changing the adhesion properties of the cells. In a recent work, Calì et al. described another kidney-related Cadherin (CDH16) as a thyroid marker and showed, in accordance with our data in human PTCs, that CDH16 repression is an early hallmark of thyroid malignant transformation [24]. The specific pattern of adhesion molecules displayed by different cell types is functional to precisely controlling the interaction of the cells with the surrounding microenvironment. The parallel observed between thyroid tumors and renal carcinomas in terms of CDH6 and CDH16 expression would suggest that these tumor types share at least a part of the mechanisms governing tumor progression.

It is known that, besides their function in connecting cells, the Cadherins (and in particular E-CAD and N-CAD) also have a signaling role by contacting downstream molecules through their cytoplasmic domains [35]. With this in mind, it is interesting to notice that the two splicing variants of CDH6 differ specifically in terms of the presence of the cytoplasmic domain, which lacks entirely in the CDH6 form. We show that both isoforms are expressed in thyroid cells. It is worth noting that the levels of CDH6-L and CDH6-S in normal thyrocytes are comparable. By contrast, CDH6-L is highly expressed and more abundant than the short form in tumor-derived cells, suggesting that the intracellular interactions of CDH6 could be functional to the role of this protein during tumor progression. No knowledge concerning a different function of the two CDH6 isoforms is currently available. As well, no data on the differential expression of these splicing forms in other tumor types have been reported. We may speculate that the two CDH6 isoforms have different abilities to transduce signals within the cells. However, further studies are needed to address this issue.

When talking about cancer, EMT means tumor invasion and metastasization. The loss of cell-cell interaction, lack of polarity, and increases in the extracellular matrix interfaces allow tumor cells to escape the rigid organization of the epithelial tissue, acquiring motility and the ability to invade other loci. While a massive amount of molecular studies has unequivocally demonstrated the relevance of these phenotypic and functional changes to the invasive properties of epithelial tumor cells, clinicians often argue that the EMT process is not visible in cancer patients, since by the time the transition is completed the epithelial cell has become a mesenchymal cell indistinguishable from the stromal components surrounding the tumor [36]. In a recent review, Klymkowsky and Savagner argue that the term EMT is often erroneously applied to distinct biological processes when, instead, it would be more correct to talk of EMT-related processes, which can vary in intensity from a transient loss of polarity to total and complete cellular reprogramming [37]. Our work seems to be in accordance with this concept, since it shows that EMT features are not restricted to a subset of invasive cells but are acquired as a consequence of the malignant transformation of the cells both in vitro and in human patients. On the other hand, the remaining ability of thyroid tumor cells to further increase some of the EMT markers (e.g. CDH6) in response to TGF-β implies an incomplete transdifferentiation of the cells. The idea that malignant transformation is accompanied by the acquisition of an EMT-related phenotype is supported by evidence that shows how somatic mutations in several oncogenes induce acquisition of EMT features [34,37-41]. Using an elegant model of 3D cultures, Jechlinger et al. have shown that loss of the epithelial phenotype in mammary cells is observed during the tumor transformation induced by forced expression of oncogenes such as Myc and mutant KRAS. The oncogene withdrawal causes regression of the tumor and the restoration of a normal epithelial phenotype in surviving cells [42]. It is reasonable to suppose that in order to sustain the uncontrolled proliferation and to escape the physical restriction imposed by the rigid organization of epithelial tissues, tumor cells need to reorganize their cellular structure by changing adhesion properties, polarity, and cytoskeleton organization in a way similar to that occurring during EMT [43].

It is not unusual for the Cadherin family members to play non-redundant functions. If we consider the EMT program as a multistep process, it is reasonable to suppose that different Cadherins play different roles in different phases of the process. Based on our expression data, it would be tempting to speculate that, unlike other mesenchymal markers widely expressed in the tumor tissues, CDH6 could be preferentially involved in the later phases of EMT more directly linked to invasiveness of the cells. Further functional data are required in order to support this hypothesis and to fully understand the function of CDH6 in the EMT program during tumor progression. Overall, the data presented in this work indicate that CDH6 is a potential mesenchymal marker of the EMT program and suggest its potential role in controlling invasiveness of thyroid tumors.

## Supporting Information

Figure S1 **CDH6 induction upon TGF-β treatment in A549 in lung cancer cell line.** qRT-PCR analysis of CDH6 expression in non-treated (NT) or TGF-β treated (5 ng/ml and 100 ng/ml) A549 lung cancer cell line. The bars represent the averaged fold change in TGF-β treated cells as compared to non-treated cells, normalized to the GAPDH levels. Error bars represent s.e.m. (n=3). All cell lines were grown at 37°C and 5% CO2.
(TIFF)

Figure S2 **Localization of CDH6 in PTC samples.** A-D) Immunohistochemistry staining (brown) of CDH6 in PTC samples. A-B) show how the CDH6 staining marks in particular cells at the invasion front of the tumor. C-D) show that CDH6 staining is localized specifically at the cell membrane. E-F) Immunohistochemistry staining (brown) of CDH6 in normal tissue surrounding the PTC samples. CDH6 expression in normal thyrocytes is barely detectable. G) Schematic representation of CDH6-L and CDH6-S isoforms. Arrows indicate primers used for the expression analysis. H-I) qRT-PCR analysis of SNAI 1 (H) and SNAI 2 (I) levels in Nthy.ori 3.1 cells non-treated (black bars) or treated with TGF-β (white bars) after transfection with RUNX2 siRNA (right) or control siRNA (left). For both control siRNA and RUNX2 siRNA-treated samples, the bars represent the relative fold change of SNAI 1 and SNAI2 after TGF-β treatment as compared to non-treated cells.
(TIFF)

Figure S3 **RUNX2 overexpression in NTHY.ORI 3.1 cells.** A) Immunofluorescence staining of SMAD2/3 in B-CPAP and TPC1 cells with or without exposure to the TGF-β inhibitor SB-431542 (24h). SMAD2/3 showed mainly a nuclear localization in untreated cells (Mock). By contrast in cells treated with the inhibitor SMAD2/3 is mainly citoplasmic. This observation demonstrated that SB-431542 blocks TGF-β signaling in our system. B) qRT-PCR analysis of RUNX2 expression levels in Nthy.ori 3.1 cells transefected with pCDNA3.1 vector (control) or with increasing amount of the pCDNA3.1 RUNX2 isoformI construct31. C) qRT-PCR analysis of CDH6 expression in Nthy.ori 3.1 cells transefected with pCDNA3.1 vector (control) or with increasing amount of the pCDNA3.1 RUNX2 isoformI construct31. CDH6 levels do not change after RUNX2 overexpression. mRNA analysis was performed 48h after transfection.
(TIFF)

Table S1 **Primers.**
(DOCX)

Table S2 **Clinical-pathological features of PTC patients.**
(DOCX)
