# Supplementary figures and images for: Cadherin 6 Is a New RUNX2 Target in TGF-β Signalling Pathway
Source: PLoS One. 2013 Sep 12;8(9):e75489. doi: 10.1371/journal.pone.0075489 (PMC3772092; doi:10.1371/journal.pone.0075489)

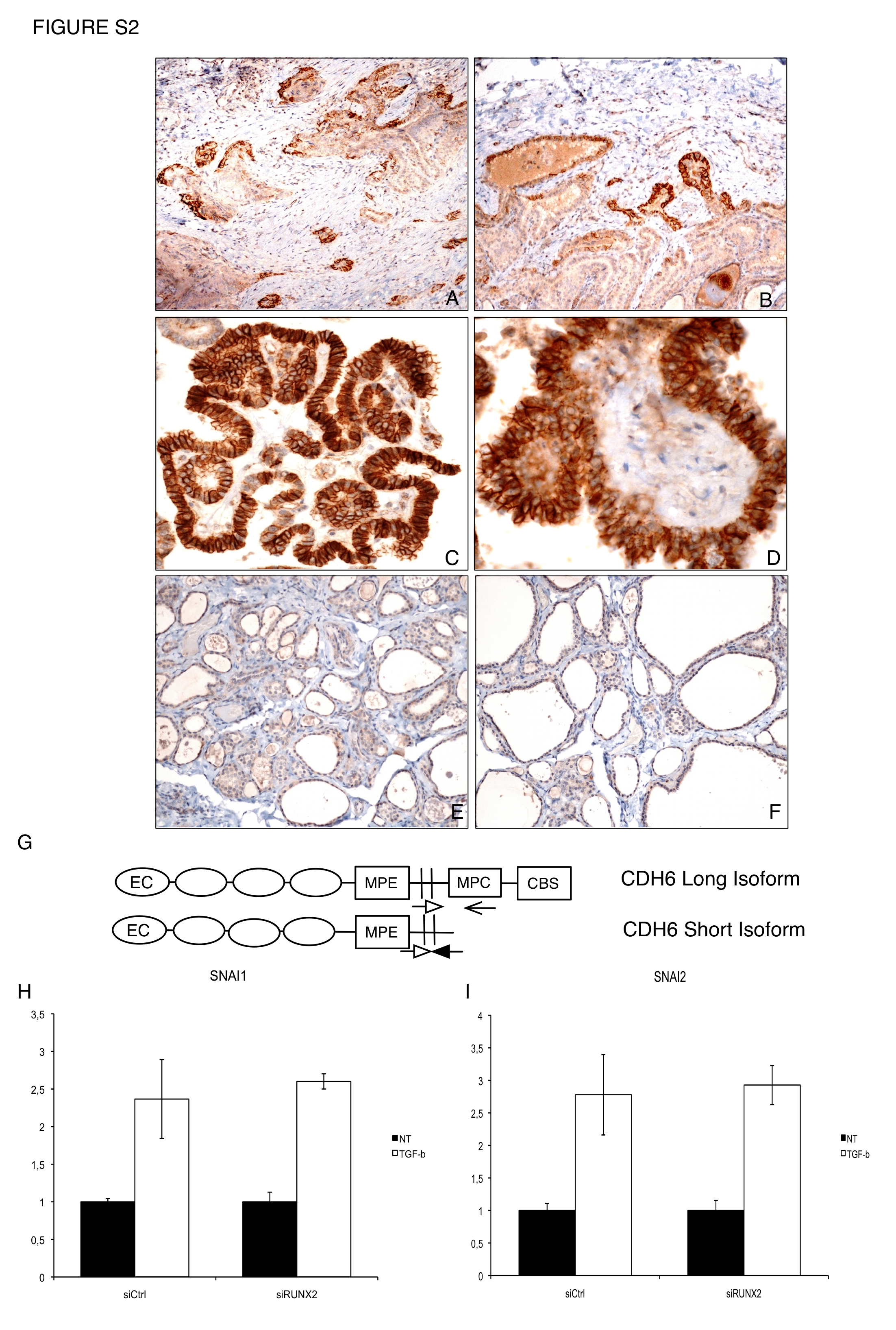

Supplement: Figure S2 — Localization of CDH6 in PTC samples. A-D) Immunohistochemistry staining (brown) of CDH6 in PTC samples. A-B) show how the CDH6 staining marks in particular cells at the invasion front of the tumor. C-D) show that CDH6 staining is localized specifically at the cell membrane. E-F) Immunohistochemistry staining (brown) of CDH6 in normal tissue surrounding the PTC samples. CDH6 expression in normal thyrocytes is barely detectable. G) Schematic representation of CDH6-L and CDH6-S isoforms. Arrows indicate primers used for the expression analysis. H-I) qRT-PCR analysis of SNAI 1 (H) and SNAI 2 (I) levels in Nthy.ori 3.1 cells non-treated (black bars) or treated with TGF-β (white bars) after transfection with RUNX2 siRNA (right) or control siRNA (left). For both control siRNA and RUNX2 siRNA-treated samples, the bars represent the relative fold change of SNAI 1 and SNAI2 after TGF-β treatment as compared to non-treated cells. (TIFF) [file pone.0075489.s002.tiff]
