# Supplementary material for: Cadherin 6 Is a New RUNX2 Target in TGF-β Signalling Pathway
Source: PLoS One. 2013 Sep 12;8(9):e75489. doi: 10.1371/journal.pone.0075489 (PMC3772092; doi:10.1371/journal.pone.0075489)
Supplement: Table S1 — Primers. (DOCX) [file pone.0075489.s004.docx]

Table S1: Primers

| E-CAD for | tgcccagaaaatgaaaaagg |
| --- | --- |
| E-CAD rev | gtgtatgtggcaatgcgttc |
| N-CAD for | ggtggaggagaagaagaccag |
| N-CAD rev | ggcatcaggctccacagt |
| CDH16 for | tgcagagctgtctgtggaag |
| CDH16 rev | cccctgacagcacgatct |
| TNC for | acctaggtctctcgcccatc |
| TNC rev | ttcagcagaattggggattt |
| VIM for | aaagtgtggctgccaagaac |
| VIM rev | agcctcagagaggtcagcaa |
| FN1 for | tccacaagcgtcatgaagag |
| FN1 rev | cgtcccagtctctgaatcct |
| CDH6 for | tcacagcccaagatccaga |
| CDH6 rev | tctgtccatatctgtgtgtcgat |
| CDH6-L for | tctggttgccatccttctgt |
| CDH6-L rev | tggaaatgatcaaaggctct |
| CDH6-S for | tctggttgccatccttctgt |
| CDH6-S rev | aatgggggaggcagataaat |
| TGFR1 for | gttccgtgaggcagagattt |
| TGFR1 rev | ctgacaccaaccagagctga |
| TGFR2 for | ctggtgctctgggaaatgac |
| TGFR2 rev | caccttggaaccaaatggag |
| SNAI1 for | ctacaaggccatgtccggacc |
| SNAI1 rev | ggaggtgggcccgcaggt |
| SNAI2 for | gcctccaaaaagccaaacta |
| SNAI2 rev | cacagtgatggggctgatg |
| ZEB1 for | tgcactgagtgtggaaaagc |
| ZEB1 rev | ttgcagtttgggcattcata |
| TWIST for | ggagtccgcagtcttacgag |
| TWIST rev | tctggaggacctggtagagg |
| ID1 for | cctcaacggcgagatcag |
| ID1 rev | cgcttcagcgacacaagat |
| RUNX2 for | gtgcctaggcgcatttca |
| RUNX2 rev | gctcttcttactgagagtggaagg |
| GAPDH for | attgggcgcctggtcac |
| GAPDH rev | aagatgtaaaccatgtagttgaggtca |
| CYPA for | GACCCAACACAAATGGTTCC |
| CYPA rev | TTTCACTTTGCCAAACACCA |
| GUSB for | TTGAGCAAGACTGATACCACCTG |
| GUSB rev | TCTGGTCTGCCGTGAACAGT |
