# Supplementary material for: Cadherin 6 Is a New RUNX2 Target in TGF-β Signalling Pathway
Source: PLoS One. 2013 Sep 12;8(9):e75489. doi: 10.1371/journal.pone.0075489 (PMC3772092; doi:10.1371/journal.pone.0075489)
Supplement: Table S2 — Clinical-pathological features of PTC patients. (DOCX) [file pone.0075489.s005.docx]

Table S2: Clinical-Pathological features of PTC patients

| **CLINICAL AND HISTOPATHOLOGICAL FEATURES** | | | | |
| --- | --- | --- | --- | --- |
|  |  |  |  |  |
| PATIENT | SEX | AGE | HISTOLOGICAL TYPE | pTN |
| PTC1 | F | 31 | PTC | T1N1 |
| PTC2 | M | 71 | PTC | T3N1 |
| PTC3 | M | 42 | PTC TCV | T2N1 |
| PTC4 | F | 24 | PTC | T1N1 |
| PTC5 | M | 78 | PTC | T3N1 |
| PTC6 | F | 71 | PTC | T2N1 |
| PTC7 | F | 26 | PTC TCV | T1N1 |
| PTC8 | F | 44 | PTC TCV | T3N1 |
| PTC9 | F | 38 | PTC | T3N1 |
| PTC10 | F | 24 | PTC | T3N1 |
| PTC11 | F | 72 | PTC | T1N1 |
| PTC12 | M | 41 | PTC | T2N0 |
| PTC13 | F | 50 | PTC | T2N0 |
| PTC14 | F | 48 | PTC TCV | T3N1 |
| PTC15 | F | 28 | PTC | T2N1 |

PTC: papillary thyroid carcinoma, classic variant

PTC TCV: papillary thyroid carcinoma, tall cell variant
